# Microbial Degradation and Valorization of Plastic Wastes

**DOI:** 10.3389/fmicb.2020.00442

**Published:** 2020-04-21

**Authors:** Jiakang Ru, Yixin Huo, Yu Yang

**Affiliations:** ^1^Department of Biology, School of Life Science, Beijing Institute of Technology, Beijing, China; ^2^Key Laboratory of Molecular Medicine and Biotherapy, Beijing Institute of Technology, Beijing, China

**Keywords:** plastic wastes, biodegradation, valorization, depolymerase, protein engineering, synthetic biology

## Abstract

A growing accumulation of plastic wastes has become a severe environmental and social issue. It is urgent to develop innovative approaches for the disposal of plastic wastes. In recent years, reports on biodegradation of synthetic plastics by microorganisms or enzymes have sprung up, and these offer a possibility to develop biological treatment technology for plastic wastes. In this review, we have comprehensively summarized the microorganisms and enzymes that are able to degrade a variety of generally used synthetic plastics, such as polyethylene (PE), polystyrene (PS), polypropylene (PP), polyvinyl chloride (PVC), polyurethane (PUR), and polyethylene terephthalate (PET). In addition, we have highlighted the microbial metabolic pathways for plastic depolymerization products and the current attempts toward utilization of such products as feedstocks for microbial production of chemicals with high value. Taken together, these findings will contribute to building a conception of bio-upcycling plastic wastes by connecting the biodegradation of plastic wastes to the biosynthesis of valuable chemicals in microorganisms. Last, but not least, we have discussed the challenges toward microbial degradation and valorization of plastic wastes.

## Introduction

Synthetic plastics, including polyethylene (PE), polystyrene (PS), polypropylene (PP), polyvinyl chloride (PVC), polyurethane (PUR), and polyethylene terephthalate (PET) (Table 1), have become fundamental to almost every aspect of our lives. According to the latest statistics of Plastics-Europe, the global yield of plastics reached 348 million tons in 2018 (Plastics Europe, 2018). China and the European Union account for 29.4 and 18.5%, ranking first and second in the world, of all the world’s plastic use, respectively (China Plastics Industry, 2017; Plastics Europe, 2018). Concomitant with the growing consumption of plastics, the generation of plastic wastes increases rapidly around the world. It is predicted that up to 26 billion tons of plastic wastes will be produced by 2050, and more than half will be thrown away into landfills and finally enter ecospheres, such as oceans and lakes, leading to serious environmental pollution (Jambeck et al., 2015; Lönnstedt and Eklöv, 2016; Geyer et al., 2017). As a result, plastic wastes have become a malevolent symbol of our wasteful society.

**TABLE 1:** Types and properties of generally used synthetic plastics.

Plastics	Abbreviation	Structure formula	T_m_ (°C)^a^	T_g_ (°C)^b^	*X*_C_ (%)^c^	Recycling codes
High-density polyethylene	HDPE	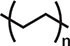	200–300	−120	80–90	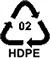
Low-density polyethylene	LDPE		160–260	−120	45–65	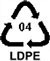
Polystyrene	PS	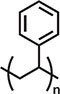	240	63–112	–	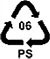
Polypropylene	PP	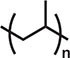	130	−10–18	60–70	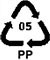
Polyvinyl chloride	PVC	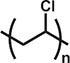	100–260	60–70	–	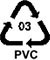
Polyethylene terephthalate	PET	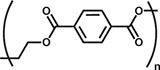	260	80	40–60	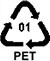
Polyester polyurethane	Polyester PUR	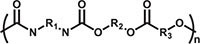	8–20 (soft)	−75 to −50 (soft) 185–205 (hard)	40–50	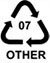
Polyether polyurethane	Polyether PUR	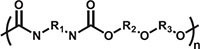	-95 (soft) 100 (hard)	−10 to 45 (soft) 190–240 (hard)		

*^*a*^T_*m*_, melting temperature. ^*b*^T_*g*_, glass transition temperature. ^*c*^X_*C*_, crystallinity.*

The current methods for disposing of plastic wastes mainly include landfilling, incineration, and mechanical and chemical recycling (Peng et al., 2018). In most countries, especially the developing countries, landfilling is the major method for plastic wastes disposal due to its operability and low cost. However, the accumulated plastic wastes have occupied a great amount of land. Incineration of plastic wastes can reduce the demand of landfills and recover heat energy, but we also need to reduce the environmental effects of secondary pollutants generated from the incinerating process, such as dioxins, carbon monoxide, nitrogen oxides, and so on. Although mechanical recycling has become the primary recycling method and is applied for reusing thermoplastic wastes, the properties of most recycled materials are significantly compromised after a number of processing cycles, and the resulting commercial values are thus limited. As an alternative, chemical recycling can recover the monomers and other chemicals from plastic wastes, but its success relies on the affordability of processes and the efficiency of catalysts (Rahimi and García, 2017). Nowadays, it is reported that only 9 and 12% of global plastic wastes is recycled and incinerated, while up to 79% is discarded into landfills or the natural environment, indicating that there is a great need for exploring innovative recycling methods to dispose of plastic wastes (Garcia and Robertson, 2017; Geyer et al., 2017).

**FIGURE 1:**
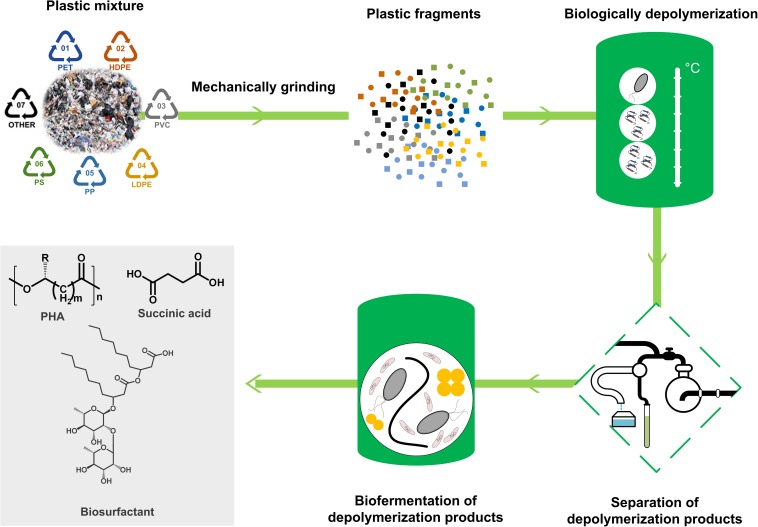
The basic conception of bio-upcycling plastic wastes. A mixture of a variety of plastic wastes will be firstly mechanically grinded and biologically depolymerized by plastic-degrading microorganisms and enzymes. Then, the depolymerization products will be separated from the culture and utilized as feedstocks for microbial fermentation to produce chemicals with high value, such as polyhydroxyalkanoate (PHA), succinic acid, and biosurfactant.

In recent years, a number of studies have reported that several microorganisms and enzymes are capable of degrading synthetic plastics. Although numerous reviews and viewpoints on the topic of biodegradation of plastic have been published, they have mainly focused on the biodegradation of a single kind of plastic, such as PE (Restrepo-Flórez et al., 2014), PS (Ho et al., 2018), PP (Arutchelvi et al., 2008), PUR (Cregut et al., 2013; Peng et al., 2018; Magnin et al., 2019c), and PET (Wei and Zimmermann, 2017a; Kawai et al., 2019; Taniguchi et al., 2019). A comprehensive review into biodegradation of all main kinds of plastic is necessary (Wei and Zimmermann, 2017b). Moreover, a review focusing on not only the biodegradation but also the biological upcycling of plastic wastes is even more attractive (Wierckx et al., 2015; Salvador et al., 2019; Blank et al., 2020). In this review, we have summarized the microorganisms and enzymes that have been proven to be capable of degrading plastics, such as PE, PS, PP, PVC, PUR, and PET, as well as the microbial metabolic pathways of the plastic depolymerization products and the current attempts toward utilization of these products as feedstocks for microbial valorization. Based on the above understandings, we have attempted to develop a biologically upcycling conception for plastic wastes through building a metabolic link between biodegradation of plastic wastes and biosynthesis of valuable chemicals in microorganisms (Figure 1). Finally, we have discussed the existing knowledge gaps and challenges facing microbial degradation and valorization of plastic wastes.

## Microbial Degradation of Synthetic Plastics

A number of microorganisms capable of degrading polyolefins (PE, PS, and PP), PVC, PUR, and PET have been isolated from the open environment, such as the soil of a plastic-dumping site, waste of mulch films, marine water, soil contaminated by crude oil, sewage sludge, landfills, and the guts of plastic-eating worms (Tables 2–7). The screening of plastic-degrading microorganisms is crucial for identifying the depolymerases and other key enzymes involved in plastic degradation.

**TABLE 2:** Bacteria, fungi, and enzymes associated with polyethylene (PE) biodegradation.

Strain/Enzyme	Isolated source	Tested PE	Incubation time, d	Weight loss, %	Molecular weight	Degradation products	References
*Rhodococcus ruber* C208	Soil of disposal site	LDPE film	30	4	–	–	Orr et al., 2004
*Bacillus sphericus* Alt; *Bacillus cereus* BF20	Marine water	LDPE film	180	2.5–10	–	–	Sudhakar et al., 2008
*Arthrobacter* sp. GMB5; *Pseudomonas* sp. GMB7	Plastic waste dumpsites	HDPE film	30	12–15	–	–	Balasubramanian et al., 2010
*Pseudomonas* sp. E4	Soil	LMWPE	80	–	–	–	Yoon et al., 2012
*Pseudomonas* sp. AKS2	Waste dumping soil	LDPE film	45	5	–	–	Tribedi and Sil, 2013
*Bacillus subtilis* H1584	Marine water	LDPE film	30	1.75	–	–	Harshvardhan and Jha, 2013
*Enterobacter asburiae* YT1; *Bacillus* sp. YP1	Gut of waxworm	LDPE film	60	6–11	Decreased	Detected	Yang et al., 2014
*Serratia marcescens*	Ground soil	LLDPE film	70	36	–	–	Azeko et al., 2015
*Achromobacter xylosoxidans*	Soil	HDPE film	150	9.38	–	–	Kowalczyk et al., 2016
*Zalerion maritimum*	Marine environment	PE pellets	28	–	–	–	Paço et al., 2017
*Phormidium lucidum*; *Oscillatoria subbrevis*	Domestic sewage water	LDPE film	42	–	–	–	Sarmah and Rout, 2018
*Alcanivorax borkumensis*	Mediterranean Sea	LDPE film	7	3.5	–	–	Delacuvellerie et al., 2019
manganese peroxidase	*Phanerochaete chrysosporium*	PE film	12	–	Decreased	–	Iiyoshi et al., 1998
soybean peroxidase	Soybean	HDPE film	2 h	–	–	–	Zhao et al., 2004
laccase	*Rhodococcus ruber* C208	LDPE film	30	2.5	Decreased	–	Santo et al., 2013
*alkB* gene	*Pseudomonas* sp. E4	LMWPE sheet	80	19.3	–	–	Yoon et al., 2012
*alkB1*, *alkB2* gene	*Pseudomonas aeruginosa* E7	LMWPE film	50	19.6–27.6	–	–	Jeon and Kim, 2016a

### PE

As early as the 1970s, Albertsson carried out an experiment on microbial degradation of ^14^C-labeled PE (average weight molecular weight of 300,000 Da) by using three different soil microbiotas as inocula (Albertsson, 1978). In terms of the release of ^14^CO_2_, the microbial degradation rate of PE was calculated to be in the range of 0.36–0.39% after 2 years (Albertsson, 1978). When the ^14^C-labeled PE was extracted with cyclohexane to get rid of its low molecular weight components (average weight molecular weight of 1,000 Da), the microbial degradation rate dropped to 0.16% (Albertsson, 1980). Therefore, it was concluded that the release of ^14^CO_2_ was mainly derived from the microbial degradation of the low molecular weight PE fraction, which was similar to the microbial degradation of straight-chain *n*-alkanes (Albertsson, 1980). After that, Kawai et al. claimed that the upper limit of molecular weight for PE degradation by microorganisms was about 2,000 Da based on the results of a numerical simulation (Kawai et al., 1999, 2002, 2004; Watanabe et al., 2003, 2004).

Although the high molecular weight was considered as a key factor impeding the microbial degradation of PE, the physicochemical pretreatments, including UV irradiation (Albertsson et al., 1987; Albertsson and Karlsson, 1988, 1990), chemical oxidizing agents (Brown et al., 1974), and thermo-oxidation (Lee et al., 1991), could facilitate the microbial degradation of long-chain PE since these pretreatments led to the depolymerization of long-chain PE as well as the formation of low molecular weight products (Albertsson et al., 1995, 1998; Erlandsson et al., 1998; Hakkarainen and Albertsson, 2004). Consequently, it was assumed that the environmental degradation of long-chain PE could be achieved by the synergistic actions of photo- or thermo-oxidation and the biological activity of microorganisms (Hakkarainen and Albertsson, 2004).

Nevertheless, it was intriguing to figure out whether the long-chain PE (molecular weight > 2,000 Da) could be degraded by microorganisms from nature. A number of strains capable of degrading un-pretreated PE have been isolated from a variety of environments, including mulch films, marine water, soil contaminated by crude oil, sewage sludge, and landfills (Table 2; Orr et al., 2004; Sivan et al., 2006; Sudhakar et al., 2008; Balasubramanian et al., 2010; Yoon et al., 2012; Tribedi and Sil, 2013; Harshvardhan and Jha, 2013; Yang et al., 2014; Azeko et al., 2015; Kowalczyk et al., 2016; Paço et al., 2017; Sarmah and Rout, 2018; Delacuvellerie et al., 2019). Some of these strains showed the ability to utilize un-pretreated PE as a carbon source based on the characterizations of biofilm formation on PE films, weight loss of PE materials, surface deterioration, and changes in the mechanical and thermal properties of PE (Table 2). For example, it was reported that the weight loss of un-pretreated PE degraded by a strain *Serratia marcescens* reached 36% in an incubation period of 70 days (Azeko et al., 2015). Moreover, two cyanobacteria, *Phormidium lucidum* and *Oscillatoria subbrevis*, exhibited the capability of degrading 30% of the initial weight of tested PE over a 42-day period (Sarmah and Rout, 2018). However, these promising reports of PE degradation based on weight loss are less convincing since there is no additional evidence to support that the weight loss is caused by the degradation of the long-chain PE other than the low molecular weight components in PE.

Notably, a few studies reported that the waxworms, possessing an inherent ability to feed on and digest beeswax, could chew, and eat PE films (Yang et al., 2014; Bombelli et al., 2017; Chalup et al., 2018; Kundungal et al., 2019). The biodegradation of PE has been detected through contact with the homogenate of the waxworm *Galleria mellonella* (Bombelli et al., 2017) or after passage through the gut of the lesser waxworm *Achroia grisella* (Kundungal et al., 2019), according to the changes in chemical compositions characterized by the analyses of Fourier transform infrared spectroscopy (FTIR) and nuclear magnetic resonance (NMR). However, further investigations are necessitated in order to determine whether the depolymerization of PE has occurred in the waxworm gut.

As intestinal microbial symbionts have been recognized as indispensable for the digestion of insects (Engel and Moran, 2013), we have hypothesized that the microbial symbionts in the waxworm gut also play an important part in the degradation of PE (Yang et al., 2014). Two bacterial strains, *Enterobacter asburiae* YT1 and *Bacillus* sp. YP1, were isolated from the gut of waxworm *Plodia interpunctella*, and their PE-degrading capability was documented within a limited incubation period of 60 days based on the characterizations of biofilm formation, changes in the PE physical properties (tensile strength and surface topography), chemical structure (hydrophobicity and appearance of carbonyl groups), molecular weight (accompanied by the formation of daughter products), and weight loss (Table 2). These findings indicated that the bacteria from waxworms could be a promising source for the further screening PE-degrading microbes (Yang et al., 2014; Yang et al., 2015a).

Although a diverse range of PE-degrading microbes has been reported, only four microbial enzymes have been shown to be responsible for PE degradation (Table 2). Iiyoshi et al. (1998) found that manganese peroxidase (MnP), from lignin-degrading fungi *Phanerochaete chrysosporium*, could decrease the tensile strength and average molecular weight of PE film. Zhao et al. (2004) also found that the combination of soybean peroxidase (SBP) and hydrogen peroxide could oxidize the surface of PE film and diminish the surface hydrophobicity. Santo et al. (2013) showed that the extracellular laccase secreted by the PE-degrading bacterium, *Rhodococcus ruber* C208, could oxidize the PE films to generate carbonyl groups and decrease the molecular weight. While these past studies have identified the above peroxidase and laccase to be capable of catalyzing the degradation of PE, their catalytic mechanisms in the process of microbial degradation of PE remained unclear. In addition, three alkane hydroxylase genes, *alkB, alkB1*, and *alkB2*, were cloned in *Escherichia coli* and the resulting recombinant strains were found to be able to degrade low molecular-weight PE (Table 2; Yoon et al., 2012; Jeon and Kim, 2015, 2016a). These results indicated that the *alkB*, *alkB1*, or *alkB2* played a key role in the degradation of low molecular-weight PE. Additionally, a recent study based on quantum mechanics calculations also suggested that the enzymatic cleavage of carbon–carbon bonds of polyolefins (PE and PS) by oxidases or oxygenases was possible (Xu et al., 2019). However, future efforts are required to characterize the biochemical functions of the oxidases or oxygenases, such as the enzymes encoded by the genes *alkB*, *alkB1*, or *alkB2*, within the biodegradation of PE.

### PS

Guillet et al. (1974) first used two types of ^14^C-PS (α- and β-^14^C) as substrates to assess microbial degradation of PS in both soil and activated sewage sludge, and they showed that less than 0.01% could be degraded to ^14^CO_2_ in the course of 8 weeks. Afterward, ^14^C-labeled PS was also used as a substrate to determine the degradation of PS by soil microbiota, 17 lignin-degrading fungi, and five mixed floras (Sielicki et al., 1978; Kaplan et al., 1979). According to the release of ^14^CO_2_, the degradation rate was only 1.5∼3.0% during 16 weeks, up to 0.24% within 5 weeks, and 0.04∼0.57% within 11 weeks (Sielicki et al., 1978; Kaplan et al., 1979).

Besides the mixed flora, researchers have also tried to isolate PS-degrading microbes from different environment samples (Table 3). Eisaku et al. (2003) reported that three soil microorganisms, *Xanthomonas* sp., *Sphingobacterium* sp., and *Bacillus* sp. STR-YO, could degrade PS. Mor and Sivan (2008) found that an actinomycete, *Rhodococcus ruber* C208, was able to utilize PS as its sole carbon source to grow, and this led to a weight loss of 0.8% within 8 weeks. In addition, three fungi and three bacteria were isolated from the soil-buried expanded PS films, and they could adhere and grow on PS (Table 3; Atiq et al., 2010; Atiq, 2011). However, the reported biodegradation rates of PS by these strains was quite low, and there was no evidence of changes in either the physical or chemical properties of its long-chain PS molecules after microbial degradation.

**TABLE 3:** Bacteria, fungi, and enzymes associated with polystyrene (PS) biodegradation.

Strain/Enzyme	Isolated source	Tested PS	Incubation time, d	Weight loss, %	Molecular weight	Degradation products	References
*Xanthomonas* sp.; *Sphingobacterium* sp.; *Bacillus* sp. STR-YO	Field soil	PS film	8	40–56	–	–	Eisaku et al., 2003
*Rhodococcus ruber* C208	Soil of disposal site	PS film	56	0.8	–	–	Mor and Sivan, 2008
*Microbacterium* sp. NA23; *Paenibacillus urinalis* NA26; *Bacillus* sp. NB6; *Pseudomonas aeruginosa* NB26	Soil buried expanded PS film	PS film	56	–	–	Detected	Atiq et al., 2010
*Rhizopus oryzae* NA1; *Aspergillus terreus* NA2; *Phanerochaete chrysosporium* NA3	Soil buried expanded PS film	PS film	56	–	Increased	Detected	Atiq, 2011
*Exiguobacterium* sp. YT2	Mealworm’s gut	PS film	60	7.5%	Decreased	Detected	Yang et al., 2015c
hydroquinone peroxidase	*Azotobacter beijerinckii* HM121	PS film	20 min	–	Decreased	Detected	Nakamiya et al., 1997

Extraordinarily, mealworms (larvae of *Tenebrio molitor*) were reported to be able to eat and rapidly degrade up to 50% of ingested Styrofoam (trade name of PS foam) during 24 h, and this was supported by the change in chemical composition, reduction in molecular weight, and the isotopic trace after passage through the intestinal tract (Yang et al., 2015b). With the same protocols, the PS-degrading capability was also documented in a broader range of mealworms from 12 different locations worldwide, indicating that PS degradation in mealworms is ubiquitous (Yang et al., 2018). This discovery also inspired researchers to explore more insect species, such as dark mealworms (*Tenebrio obscurus*) (Peng et al., 2019) and superworms (*Zophobas atratus*) (Yang et al., 2020), that also could eat and degrade PS.

We wondered whether the microbial symbionts associated with mealworms and superworms contributed to the degradation of PS. While the gut microbial symbionts were suppressed with antibiotics, the PS-degrading capacity of mealworms or superworms was impaired. This result indicated that gut microbial symbionts played an important role in the biodegradation of ingested Styrofoam (Yang et al., 2015c, Yang et al., 2020). Furthermore, one strain of *Exiguobacterium* sp. YT2, isolated from the gut of *Tenebrio molitor*, was proven to be capable of degrading 7.5% weight of PS *in vitro* within 60 days, while the decrease in molecular weight of the residual PS pieces and the release of water-soluble daughter products were also detected (Yang et al., 2015c; Table 3). At the time of writing, more bacteria have been isolated from the gut of plastic-eating mealworms or superworms, and their potential for PS degradation is still under assessment (Xia et al., 2019).

With respect to the PS-degrading enzymes, only hydroquinone peroxidase, secreted by a lignin-degrading bacterium *Azotobacter beijerinckii* HM121, was able to depolymerize PS into low molecular products in the presence of non-aqueous medium of dichloromethane (Nakamiya et al., 1997).

### PP

In 1993, microbial degradation of PP was firstly assessed by cultures enriched from sandy soils containing PE wastes (Cacciari et al., 1993). After an incubation period of 175 days, the amount of degradation products, which were extracted with methylene chloride, accounted for 40% of the initial weight of tested PP. However, 90% of the extracted products were identified as aromatic esters, which were derived from the plasticizers, a chemical added especially into plastic to adjust the flexibility, workability, or stretchability. Meanwhile, only 10% of the extracted products were identified as hydrocarbons (C_10_H_22_ to C_31_H_64_) that may be derived from the degradation of PP itself. This result indicated that the plasticizers, other than the PP itself, were prone to be degraded by the sandy soil microorganisms (Cacciari et al., 1993).

From that time on, several microorganisms from different environmental samples have been tested for their potential to degrade PP (Table 4). For example, when PP films were incubated with soil microbiota from a plastic-dumping site, 0.4% weight loss and 33% increase in the crystallinity of residual PP were observed after 12 months, implying that the amorphous parts of PP could be degraded by soil microbiota (Arkatkar et al., 2009). Additionally, it was found that three bacteria and two fungal strains (Table 4), isolated from the soil of a plastic-dumping site, could utilize PP as their carbon source for growth and degrade 0.05–5% of PP after incubation for 12 months (Arkatkar et al., 2010; Jeyakumar et al., 2013). Mixed consortia of four bacterial isolates, from waste management landfills and sewage treatment plants, could also degrade the PP strips and pellets with a weight loss of 44.2–56.3% after 140 days (Skariyachan et al., 2018). Moreover, two marine bacteria of *Bacillus* sp. strain 27 and *Rhodococcus* sp. strain 36, isolated from mangrove environments, were also able to grow in aqueous synthetic media containing PP microplastics and caused a weight loss of 4.0–6.4% after 40 days (Auta et al., 2018). However, it is hard to determine whether the weight loss caused by the reported microbes above was attributed to the depolymerization of the long-chain PP or the degradation of the low molecular weight components, as the analyses of changes in molecular weight were absent.

**TABLE 4:** Bacteria, fungi, and enzymes associated with polypropylene (PP) biodegradation.

Strain/Enzyme	Isolated source	Tested PP	Incubation time, d	Weight loss, %	Molecular weight	Degradation products	References
*Pseudomonas stutzeri; Bacillus subtilis; Bacillus flexus*	Plastic-dumping site	PP film	365	–	–	Detected	Arkatkar et al., 2010
*Phanerochaete chrysosporium*; *Engyodontium album*	Plastic-dumping site	PP film	365	4–5	–	Detected	Jeyakumar et al., 2013
*Stenotrophomonas panacihumi*	Soil of waste storage yard	PP film	90	–	Increased	–	Jeon and Kim, 2016b
*Aneurinibacillus aneurinilyticus*; *Brevibacillus agri*; *Brevibacillus* sp.; *Brevibacillus brevis*	Landfills and sewage	PP film and pellets	140	22.8–27.0	–	Detected	Skariyachan et al., 2018
*Bacillus* sp. strain 27; *Rhodococcus* sp. strain 36	Mangrove environments	PP microplastic	40	4–6.4	–	–	Auta et al., 2018

A mesophilic strain, *Stenotrophomonas panacihumi* PA3-2, isolated from the soil of an open storage yard for municipal solid waste, was reported to be able to degrade two kinds of low molecular weight PP (Mn: 2,800, 3,600 Da) and one high molecular weight PP (Mn: 44,000 Da) with a biodegradability of 12.7–20.3% in terms of CO_2_ release and an increase in the molecular weight after 90 days (Jeon and Kim, 2016b). The results indicated that this strain could only degrade the low molecular weight fractions rather than the long-chain PP.

Until now, there are no enzymes reported to be capable of degrading PP, and little knowledge is available for the mechanism of microbial degradation of PP (Arutchelvi et al., 2008). However, similar to PE, it was found that the physicochemical pretreatments, including γ-irradiation (Alariqi et al., 2006), UV irradiation (Jeyakumar et al., 2013), thermo-oxidation (Jeyakumar et al., 2013), and blend with degradable additives, could facilitate the microbial degradation of PP (Jeyakumar et al., 2013; Jain et al., 2018).

### PVC

Among all main kinds of synthetic plastics, PVC possesses the highest proportion of plasticizer (up to 50%). As plasticizers can be utilized by many fungi or bacteria as sources of nutrient carbons, plasticized PVC is usually susceptible to fungal or bacterial attack (Berk, 1950; Berk et al., 1957; Bessems, 1988; Kurane, 1988; Gumargalieva et al., 1999; Webb et al., 1999). For instance, a number of plasticized PVC bathroom items, such as bathtub lids, bath mats, and shower curtains, were found to be damaged by a variety of fungi (Table 5; Moriyama et al., 1993). Several fungal isolates (Table 5) from various environmental samples, such as atmosphere (Webb et al., 2000), plasticized PVC sheets buried in the grassland soil (Sabev et al., 2006; Ali et al., 2014), and plastic wastes disposal sites (Khatoon et al., 2019), also exhibited the ability to deteriorate the plasticized PVC. In addition, a number of bacterial strains (Table 5), isolated from garden soil, landfill leachate, waste disposal sites, and marine environments, have also been reported to be able to degrade the plasticized PVC (Nakamiya et al., 2005; Latorre et al., 2012; Anwar et al., 2016; Kumari et al., 2019; Giacomucci et al., 2019). However, these abovementioned plasticized PVC-degrading microorganisms just metabolized a component of the plasticizer [such as bis (2-ethylhexyl) phthalate, DEHP] rather than the backbone of PVC. Microorganisms capable of degrading both PVC and plasticizers have not been discovered so far. Thus, the key enzymes involved in the microbial degradation of PVC are still unknown.

**TABLE 5:** Bacteria, fungi, and enzymes associated with polyvinyl chloride (PVC) biodegradation.

Strain/Enzyme	Isolated source	Tested PVC	Incubation time, d	Weight loss,%	Molecular weight	Degradation products	References
*Alternaria* sp. TOF-46	Japanese bathrooms	Plasticized PVC rim	180	–	–	–	Moriyama et al., 1993
*Poliporus versicolor; Pleurotus sajor caju*	Lignocellulosic waste	PVC film	30	–	–	Detected	Klrbas et al., 1999
*Aureobasidium pullulans*	Leaf/wood surfaces	Plasticized PVC	7	–	–	–	Webb et al., 1999
*Aspergillus niger*	PVC wires	Plasticized PVC film	365	–	–	–	Gumargalieva et al., 1999
*Aureobasidium pullulans*	Atmosphere	Plasticized PVC film	42	3.7	–	–	Webb et al., 2000
*Penicillium janthinellum*	PVC buried in soil	Plasticized PVC sheet	300	–	–	–	Sabev et al., 2006
*Mycobacterium* sp. NK0301	Garden soil	Plasticized PVC film	3	–	–	Detected	Nakamiya et al., 2005
*Chryseomicrobium imtechense*; *Lysinibacillus fusiformis*; *Acinetobacter calcoaceticus*; *Stenotrophomonas pavanii*	Landfill leachate	Plasticized PVC curtain	34	–	–	–	Latorre et al., 2012
*Phanerochaete chrysosporium*; *Lentinus tigrinus*; *Aspergillus niger*; *Aspergillus sydowii*	PVC film buried in soil	PVC film	300	–	Decreased	Detected	Ali et al., 2014
*Acanthopleurobacter pedis*; *Bacillus cereus*; *Pseudomonas otitidis*; *Bacillus aerius*;	Plastic disposal sites	PVC film	90	–	Decreased	Detected	Anwar et al., 2016
*Bacillus* sp. AIIW2	Marine	Un-plasticized PVC film	90	0.26	–	Detected	Kumari et al., 2019
*Phanerocheate chrysosporium*	Plastic disposal site	PVC film	28	31	–	Detected	Khatoon et al., 2019
*Pseudomonas citronellolis*	Soil	Plasticized PVC film	45	13	Decreased	–	Giacomucci et al., 2019

In future screening experiments, it is important to characterize the ability of strains to depolymerize the long-chain molecules of PVC by using virgin plastic in which low molecular weight components (monomers, oligomers, and plasticizers) were extracted by use of a suitable solvent or determining the decrease in the average molecular weight and the broadening of the molecular weight distribution of the residues after degradation.

### PUR

PUR is the universal nomenclature for the plastic derived from the condensation of polyisocyanates and polyols with the linkages of intramolecular urethane bonds (Table 1). Depending on the chemical structures of the polyols used, PUR synthesized from polyester polyol is designated as polyester PUR, while that synthesized from polyether polyol is termed as polyether PUR.

In 1968, the initial research into microbial degradation of PUR was made by Darby and Kaplan. They found that seven fungi can grow on the surface of solid polyester PUR (Table 6; Darby and Kaplan, 1968). Since then, a number of fungi have been proven to be able to degrade polyester PUR (Table 6; Crabbe et al., 1994; Cosgrove et al., 2007; Russell et al., 2011; Mathur and Prasad, 2012; Álvarez-Barragán et al., 2016; Khan et al., 2017; Osman et al., 2017; Magnin et al., 2019a). In addition to fungi, many bacteria also have been demonstrated to be capable of degrading polyester PUR (Table 6; Kay et al., 1991, 1993; Nakajima-Kambe et al., 1995, 1997; Howard and Blake, 1998; Ii et al., 1998; Howard et al., 1999, 2001a,b; Rowe and Howard, 2002; Oceguera-Cervantes et al., 2007; Gautam et al., 2007; Nair and Kumar, 2007; Shah et al., 2008, 2013a,b, 2016; Howard and Burks, 2012; Peng et al., 2014; Nakkabi et al., 2015a, b; Pérez-Lara et al., 2016).

**TABLE 6:** Bacteria, fungi, and enzymes associated with polyurethane (PUR) biodegradation.

Strain/Enzyme	Isolated source	Tested PUR	Incubation time, d	Weight loss, %	Molecular weight	Degradation products	References
*Chaetomium globosum*	Soil	Polyester/polyether PUR film	21	–	–	–	Darby and Kaplan, 1968
*Curvularia senegalensis*	Soil	Impranil DLN	7	–	–	–	Crabbe et al., 1994
*Geomyces pannorum*	Acidic soil	Impranil DLN	150	–	–	–	Cosgrove et al., 2007
*Alternaria* sp. PURDK2	Environment	Polyether PUR film	70	27.5	–	Detected	Matsumiya et al., 2010
*Pestalotiopsis microspora*	Plant stems	Impranil DLN,	14	–	–	–	Russell et al., 2011
*Aspergillus flavus*	Plastic disposal sites	Polyester PUR film	30	60.6	–	–	Mathur and Prasad, 2012
*Cladosporium tenuissimum*	Garden soil	Impranil DLN; polyether varnish	14	65	–	Detected	Álvarez-Barragán et al., 2016
*Aspergillus tubingensis*	Waste disposal site	Polyester PUR beads	20	–	–	–	Khan et al., 2017
*Aspergillus* sp. S45	Waste-dumping site	Polyester PUR film	28	15–20	–	Detected	Osman et al., 2017
*Penicillium* sp.	PUR wastes	Impranil DLN; polyester/polyether PUR film	60	8.9	Decreased	–	Magnin et al., 2019a
*Corynebacterium* sp., BI2; *Pseudomonas aeruginosa*	Soil	Polyester PUR foam	84	1.2–17.7	–	–	Kay et al., 1991
*Comamonas acidovorans*	Soil	Polyester PUR film	7	–	–	Detected	Nakajima-Kambe et al., 1995
*Bacillus* sp.	Soil	Impranil DLN	4	–	–	–	Ii et al., 1998
*Pseudomonas fluorescens*	Soil	Impranil DLN	ND	–	–	–	Howard and Blake, 1998
*Pseudomonas chlororaphis*	Soil	Impranil DLN	ND	–	–	–	Howard et al., 1999
*Bacillus subtilis*	Soil	Impranil DLN	ND	–	–	–	Rowe and Howard, 2002
*Acinetobacter gerneri*	Soil	Impranil DLN	ND	–	–	–	Howard and Burks, 2012
*Alicycliphilus* sp. *BQ1*	Decomposed soft foam	Polyester PUR film	100	–	–	Detected	Oceguera-Cervantes et al., 2007
*Bacillus pumilus*	PUR-contaminated water	Impranil DLN	3	–	–	–	Nair and Kumar, 2007
*Pseudomonas chlororaphis*	Soil	Ester PUR foam	12	–	–	–	Gautam et al., 2007
*Bacillus* sp. AF8; *Pseudomonas* sp. AF9; *Micrococcus* sp. 10; *Arthrobacter* sp. AF11; *Corynebacterium* sp. AF12	Soil	Polyester PUR film	28	–	–	–	Shah et al., 2008
*Bacillus subtilis*; *Pseudomonas aeruginosa*	Soil	Polyester PUR pellets	20	–	–	Detected	Shah et al., 2016
*Pseudomonas putida*	Soil	Impranil DLN	8	–	–	–	Peng et al., 2014
*Bacillus safensis*	Cedar wood	Impranil DLN;	7	–	–	–	Nakkabi et al., 2015a, b
*Aspergillus niger*; *Cladosporium herbarum*	Natural humid conditions	Polyether PUR foam	70	–	–	–	Filip, 1979
*Staphylococcus epidermidis*	An intravenous catheter	Polyether PUR film	30	–	–	–	Jansen et al., 1991
*Alternaria tenuissima*	Infected leaves	Polyether PUR film	60	–	–	–	Oprea et al., 2018
*Pseudomonas denitrificans, Pseudomonas fluorescens, Bacillus subtilis, Yarrowia lipolytica*	Soil	Polyether PUR film	150	2.8–10.5	–	–	Stepien et al., 2017
esterase	*Curvularia senegalensis*	Impranil DLN	21	–	–	–	Crabbe et al., 1994
pudA	*Comamonas acidovorans*	Polyester PUR film	2	–	–	–	Akutsu et al., 1998
lipase	*Bacillus subtilis*	Impranil DLN	1	–	–	–	Rowe and Howard, 2002
pulA	*Pseudomonas fluorescens*	Impranil DLN	ND	–	–	–	Ruiz and Howard, 1999
pueA	*Pseudomonas chlororaphis*	Impranil DLN	6 h	–	–	–	Stern and Howard, 2000
pueB	*Pseudomonas chlororaphis*	Impranil DLN	20 h	–	–	–	Howard et al., 2001b
LC cutinase; TfCut2; Tcur1278; Tcur0390	Compost metagenomic library; *Thermobifida fusca*	Impranil DLN; polyester PUR cubes	100 h	0.3–3.2	decreased	–	Schmidt et al., 2017
Esterase E3576	Protéus (France)	Polyester/polyether PUR film	51	33	–	Detected	Magnin et al., 2019b

With regard to polyether PUR (Table 6), it was much less susceptible to microbial degradation in comparison to the polyester PUR (Darby and Kaplan, 1968). Notwithstanding, in 1979, Filip observed growth of *Aspergillus niger* and *Cladosporium herbarum* in shake cultures with polyether PUR resilient foam as the sole nutrient source (Filip, 1979). Afterward, Jansen et al. isolated a strain of *Staphylococcus epidermidis* KH11 from an infected catheter and demonstrated its capacity to utilize polyether PUR in the absence of any organic nutrients (Jansen et al., 1991). In 2010, a fungus, *Alternaria* sp. PURDK2, was reported to be able to degrade 27.5% of the weight of tested polyether PUR foam in the Luria-Bertani (LB) glucose agar after 70 days. Furthermore, this fungus was also capable of degrading two small molecule analogs of PUR, ethylphenylcarbamate (EPC) and diphenylmethane-4,4′-dibutylurea (D-MDI), into aniline and ethanol, indicating that the fungus could secret urethane-bond–degrading enzymes (Matsumiya et al., 2010). In 2016, eight fungal strains (Table 6) were showed be able to grow in mineral medium with a polyether PUR varnish as the sole carbon source and degrade 65% of solid polyether PUR foams in 50% potato dextrose broth (PDB) over 21 days (Álvarez-Barragán et al., 2016). Stepien et al. found that three bacteria and one yeast (Table 6) could degrade commercial polyether PUR films (Tecoflex^®^) and cause a weight loss of 2.8–10.5% within 5 months (Stepien et al., 2017). Oprea et al. assessed the biodegradability of pyridine-based polyether PUR elastomers by a fungus *Alternaria tenuissima*, and found that the fungus could decrease the mechanical properties and deteriorate the surface morphology after 60 days (Oprea et al., 2018).

The genes and enzymes contributing to microbial degradation of polyester PUR have been widely investigated (Table 6). In 1994, Crabbe et al. purified an esterase from a polyester PUR-degrading fungus, *Curvularia senegalensis*, and showed that this esterase can cleavage the ester bonds in the soft segments of polyester PUR (Crabbe et al., 1994). While Akutsu et al. purified a cell surface-bond polyester PUR-degrading esterase from the polyester PUR-degrading bacterium *Comamonas acidovorans* TB-35, Nomura et al. cloned a gene *pudA* encoding polyester PUR-degrading esterase in this strain (Akutsu et al., 1998; Nomura et al., 1998). Howard et al. purified a protease from *Pseudomonas fluorescens* (Vega et al., 1999), an esterase from *Comamonas acidovorans* (Allen et al., 1999), three esterases from *Pseudomonas chlororaphis* (Howard et al., 1999; Ruiz et al., 1999), and a lipase from *Bacillus subtilis* (Rowe and Howard, 2002). All the purified serine hydrolases above have the same hydrolytic capacity to emulsify polyester PUR. In addition, they also cloned a gene named *pulA* from *Pseudomonas fluorescens* (Ruiz and Howard, 1999) and two genes, *pueA* and *pueB*, from *Pseudomonas chlororaphis* (Stern and Howard, 2000; Howard et al., 2001b). These genes encoded three different esterases involved in the microbial degradation of emulsified polyester PUR by *Pseudomonas fluorescens* and *Pseudomonas chlororaphis*. In 2017, Schmidt et al. found that four polyester hydrolases, LC cutinase, TfCut2, Tcur1278, and Tcur0390, were able to degrade emulsified polyester PUR (Schmidt et al., 2017). Among these three cutinase, LC cutinase caused weight losses of up to 4.9 and 4.1% of two commercial polyester PUR elastomers of Elastollan B85A-10 and C85A-10, respectively, within a reaction time of 200 h at 70°C. Recently, an esterase (E3576), screened from 50 commercially available hydrolases, was shown to be able to hydrolyze a waterborne polyester PUR dispersion and degrade a solid polycaprolactone polyol-based polyester PUR with weight loss of 33% after 51 days (Magnin et al., 2019b). However, this esterase (E3576) cannot degrade poly(hexamethylene adipate) diol-based polyester PUR films, indicating that the chemical structures of the polyol segments significantly affect the biodegradability of polyester PUR (Kim and Kim, 1998; Magnin et al., 2019b).

Although the above reported lipases or esterases were able to rapidly degrade the emulsified polyester PUR (Impranil DLN) by cleaving the ester bonds in the polyester polyols segments, they exhibited a weak capability of degrading the solid polyester PUR substrates, such as PUR film, foam, and elastomer (Schmidt et al., 2017). The degradation products were not identified, and the biochemical mechanism was still unclear. Moreover, no specific depolymerases have been reported to be able to degrade the polyether PUR and cleave the urethane bones in both polyester and polyether PUR.

### PET

The purpose of initial efforts to find out the hydrolases capable of hydrolyzing PET was to modify the surface wettability of PET fabrics (Hsieh and Cram, 1998; Yoon et al., 2002; Gübitz and Paulo, 2003; Alisch et al., 2004; Fischer-Colbrie et al., 2004; O’Neill and Cavaco-Paulo, 2004; Zhang et al., 2004; Silva et al., 2005; Vertommen et al., 2005). In the process of enzymatic surface modification, ester linkages on the surface of PET were hydrolyzed to produce polar hydroxyl and carboxylic groups, but the inner bulk of PET was not degraded. In a recent review focusing on enzymatic degradation of PET, Kawai et al. defined those hydrolases with moderate surface-hydrolyzing capability as *PET surface-modifying enzymes* (Kawai et al., 2019). By contrast, the hydrolases with significant capability of hydrolyzing the inner bulk of PET (causing at least 10% weight loss) were termed as *PET hydrolases* (Kawai et al., 2019). Hereinafter, only the reported *PET hydrolases* were reviewed (Table 7).

**TABLE 7:** Enzymes associated with polyethylene terephthalate (PET) biodegradation.

Enzyme	Isolated source	Tested PET	Crystallinity, %	Reaction temperature, °C	Incubation time, d	Weight loss, %	References
TfH	*Thermobifida fusca*	PET bottle and pellets	9	55	21	54.2	Müller et al., 2005
HiC; PmC; PsC	*Humicola insolens; Pseudomonas mendocina; Fusarium solani*	Low-crystallinity PET film	7	70	6	97%	Ronkvist et al., 2009
LC-cutinase	Compost metagenomic library	Low-crystallinity PET film	8.4	50	7	50	Sulaiman et al., 2012
Cut190	*Saccharomonospora viridis*	Low-crystallinity PET film	8.4	63	3	27	Kawai et al., 2014
*Is*PETase	*Ideonella sakaiensis*	Low-crystallinity PET film	1.9	30	0.75	–	Yoshida et al., 2016
*Is*PETase	*Ideonella sakaiensis*	Low-crystallinity PET film	–	30	1	1	Wei et al., 2019b
TfCut2	*Thermobifida fusca*	Low-crystallinity PET chip	7	70	5	97	Wei et al., 2019a

In 2005, Müller et al. (2005) reported that a cutinase-like hydrolase TfH, from an actinomycete *Thermobifida fusca*, can effectively degrade up to 50% of the initial weight of low-crystallinity PET (lcPET, 9%) at 55°C for 3 weeks. This is the first report on the enzymatic degradation of the inner bulk of PET films that opens the door for enzymatic PET recycling in the future (Müller, 2006). Thereafter, Ronkvist et al. compared the PET-hydrolyzing activities of three cutinases from different microorganisms, *Humicola insolens* (HiC, now named *Thermomyces insolens*), *Pseudomonas mendocina* (PmC), and *Fusarium solani* (FsC), using lcPET films (7%) and high-crystallinity biaxially oriented PET films (hcPET, 35%) as substrates (Ronkvist et al., 2009). Results showed that HiC caused a 97% weight loss of lcPET film (7%) at 70°C within 96 h, while PmC or FsC only led to a weight loss up to 5%. Thus, HiC can be designated as *PET hydrolase*, while PmC and FsC should be ascribed to *PET surface-modifying enzymes*. However, the three cutinases could hardly hydrolyze the hcPET films (35%). After that, Sulaiman et al. (2012) found that a LC-cutinase, encoded by one gene from the metagenomic library of leaf-branch compost, can efficiently hydrolyze low-crystallinity PET package film (lcPET-P, 8.4%) at 50°C and generate up to 50% weight loss over 7 days. In addition, Kawai et al. found that a cutinase Cut190, from *Saccharomonospora viridis* AHK190, can hydrolyze the lcPET (7%) and lcPET-P (8.4%) at 63°C, resulting in a weight loss of 13.5 and 27.0% for lcPET and lcPET-P, respectively, over 3 days (Kawai et al., 2014). It was recently shown that the recombinant *Thermobifida fusca* cutinase TfCut2 expressed by *B. subtilis* could degrade the lcPET films (7%) with a weight loss up to 97.0% and two low-crystallinity PET samples from postconsumer packages (AP-PET, 5%; CP-PET, 6%) with maximum weight losses of 50.5 and 56.6%, respectively, within 120 h at 70°C (Wei et al., 2019a).

Remarkably, Yoshida et al. found a bacterium, *Ideonella sakaiensis* 201-F6, capable of degrading lcPET films (1.9%) at an ambient temperature, and they identified a PET-hydrolyzing enzyme, termed as *Is*PETase, from this bacterium (Yoshida et al., 2016). The *Is*PETase was heat-labile (20∼45°C) and exhibited greater PET degradation activity than the above reported *PET hydrolases* at a mesophilic temperature of 30°C (Yoshida et al., 2016; Taniguchi et al., 2019). Nevertheless, the degradation rate of lcPET film (7%) by *Is*PETase at 30°C over a 24 h-incubation period was only 1% (weight loss), which was markedly lower than that caused by the above reported *PET hydrolases* at a thermophilic temperature (50∼70°C) (Wei et al., 2019a, b). Moreover, the hydrolytic activity of *Is*PETase against lcPET films (1.9%) was obviously higher than that for hcPET films (30∼40%) (Yang et al., 2016; Yoshida et al., 2016).

Overall, the above reported *PET hydrolases* are prone to degrade the lcPET (<10%) but not the hcPET (Vertommen et al., 2005; Ronkvist et al., 2009; Yang et al., 2016; Yoshida et al., 2016; Wei et al., 2019a). The effect of different crystallinity on the enzymatic degradation could be explained by the changes in the macromolecular aggregate structures of the polymer. Polymer molecules generally pack together in a non-uniform way with a mixture of ordered regions (crystalline-like) and disordered domains (amorphous). As the polymer chains in the amorphous domains are less densely packed than those in the crystalline domains, the lcPET, comprising a high proportion of amorphous domains, is more susceptible to enzymatic degradation. However, the high-crystallinity PET (30∼40%) represents the most abundant types of postconsumer plastic, and methods for lowering the crystallinity of PET to enhance the enzymatic degradation are highly sought.

Additionally, the enzymatic hydrolytic reactions of PET are inclined to take place under the temperature close to the glass transition temperature of PET (*T*_g_, 65∼75°C). Under such a thermophilic temperature, the polymer chains in the amorphous PET domains can gain enough mobility to access the active sites of *PET hydrolases* (Ronkvist et al., 2009; Wei and Zimmermann, 2017a; Kawai et al., 2019). As a result, it suggests that efficient enzymatic degradation of PET requires thermostable PET depolymerases. Approaches of glycosylation (Shirke et al., 2018) and rational protein engineering, such as the optimization of surface salt bridge (Shirke et al., 2016), mutation of Ca^2+^ and Mg^2+^ binding sites (Then et al., 2015), introduction of disulfide bridge (Then et al., 2016), stabilization of a β6-β7 connecting loop, and extension of subsite IIc (Son et al., 2019), have been applied to improve the thermostability of these *PET hydrolases*. Notwithstanding, there is room for increasing the half-life of PET hydrolases above 65°C.

## Microbial Valorization of Plastic Wastes

The initial step of the microbial degradation process is to secrete depolymerases to break down the long-chain polymers into low molecular weight oligomers or monomers, which can be further assimilated into microbial cells or metabolized into CO_2_. According to the principle of circular economy, these depolymerization products could be exploited for the biosynthesis of high-value chemicals through specific metabolic pathways, which could be considered as a way of valorizing plastic wastes (Wierckx et al., 2015).

### From TPA, EG, and 6-Hydroxyhexanoate to Succinic Acids and Polyhydroxyalkanoate (PHA)

**FIGURE 2:**
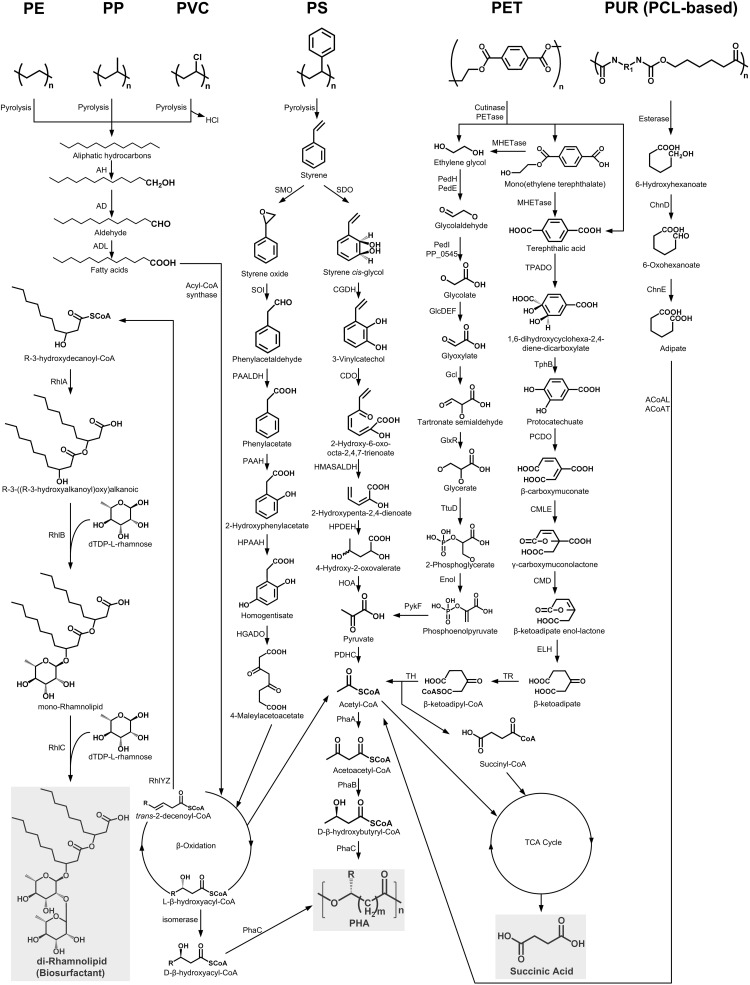
The metabolic pathways of depolymerization products of six kinds of plastics. Plastics: PE, polyethylene; PS, polystyrene; PP, polypropylene; PVC, polyvinyl chloride; PUR, polyurethane; PCL, polycaprolactone diol; PET polyethylene terephthalate. Enzymes: AH, alkane hydroxylase; AD, alcohol dehydrogenase; ALD, aldehyde dehydrogenase; RhlYZ, R-specific enoyl-CoA hydratase; RhlA, HAA synthetase; RhlB, rhamnosyltransferase 1; RhlC, rhamnosyltransferase 2; SMO, styrene monooxgenase; SOI styrene oxide isomerase; PAALDH, phenacetaldehyde dehydrogenase; PAAH, phenylacetate hydroxylase; HPAAH, 2-hydroxyphenylacetate hydroxylase; HGADO, homogentisate 1,2-dioxygenase; SDO, styrene dioxygenase; CGDH, *cis*-glycol dehydrogenase; CDO, catechol 2,3-dioxygenase; HMASALDH, 2-hydroxymuconic acid semialdehyde hydrolase; HPDEH, 2-hydroxypenta-2,4-dienoate hydratase; HOA, 4-hydroxy-2-oxovalerate aldolase; PDHC, pyruvate dehydrogenase complex; PhaA, β-ketothiolase; PhaB acetoacetyl-CoA reductase; PhaC, PHA synthase; PedH, quinoprotein alcohol dehydrogenase; PedE, quinoprotein alcohol dehydrogenase; PedI, aldehyde dehydrogenase family protein; PP_0545, aldehyde dehydrogenase family protein; GlcDEF, glycolate oxidase; Gcl glyoxylate carboligase; GlxR, tartronate semialdehyde reductase; TtuD, hydroxypyruvate reductase; PykF, pyruvate kinase; TPADO, TPA dioxygenase; TphB, 1,2-dihydroxy-3,5-cyclohexadiene-1,4-dicarboxylate dehydrogenase; PCDO, protocatechuate 3,4-dioxygenase; CMLE, β-carboxy-*cis,cis*-muconate lactonizing enzyme; CMD, β-carboxymuconolactone decarboxylase; ELH, enollactone hydrolase; TR, β-ketoadipate:succinyl-CoA transferase; TH, β-ketoadipyl-CoA thiolase; ChnD, 6-hydroxycaproate dehydrogenase; ChnE, 6-oxohexanoic dehydrogenase; ACoAL, adipate-CoA ligase; ACoAT, acetyl-CoA C-acyltransferase.

Enzymatic hydrolysis of PET could release constituent monomers ethylene glycol (EG), terephthalic acid (TPA), mono(ethylene terephthalate) (MHET), and bis(2-hydroxyethyl)TPA (BHET) by cleaving the ester bond (Ronkvist et al., 2009; Yoshida et al., 2016). Among these products, MHET could be further degraded into TPA and EG by the action of MHETase (Figure 2; Yoshida et al., 2016; Palm et al., 2019). In addition, the ester linkages in polycaprolactone polyol-based PUR (PCL-based PUR) could be hydrolyzed by an esterase (E3576) to generate 6-hydroxyhexanoate (Magnin et al., 2019b). These products could be further metabolized by specific microorganisms through different metabolic pathways (Figure 2; Brzostowicz et al., 2003; Yoshida et al., 2016).

In bacterial species, a TPA transporter is implicated in the transport of TPA into the cell (Hosaka et al., 2013). Once inside the cell, the TPA can be transformed into 1,6-dihydroxycyclohexa-2,4-diene-dicarboxylate (DCD) by the activity of the TPA dioxygenase (TPADO). DCD is further oxidized by the 1,2-dihydroxy-3,5-cyclohexadiene-1,4-dicarboxylate dehydrogenase (TphB) to form protocatechuate (PCA) (Figure 2; Wang et al., 1995; Choi et al., 2005; Sasoh et al., 2006). The PCA could be degraded by the *ortho*-, *meta*-, and *para*-cleavage pathways under the catalysis of 3,4-dioxygenase (PCDO), 4,5-dioxygenase, and 2,3-dioxygenase, respectively (Harwood and Parales, 1996). Of these, the *ortho*-cleavage pathway has been thoroughly studied. The resulting metabolite, β-carboxymuconate (CM), will eventually be converted into acetyl-CoA and succinyl-CoA, which can enter the tricarboxylic acid (TCA) cycle to generate succinic acid (Figure 2).

In 2008, Kenny et al. first isolated three microorganisms, *Pseudomonas putida* GO16, *Pseudomonas putida* GO19, and *Pseudomonas frederiksbergensis* GO23, which could utilize TPA for not only growth but also accumulation of medium chain length PHA (mclPHA). Subsequently, they used the TPA fraction from PET pyrolysis as the feedstock for microbial production of mclPHA by these *Pseudomonas* species. The maximal production rate of PHA reached approximately 8.4 mg⋅l^–1^⋅h^–1^ (Kenny et al., 2008). When TPA and glycerol waste from biodiesel manufacture were co-supplied to *Pseudomonas putida* GO16 in a fed-batch bioreactor, the production rate of PHA reached approximately 108.8 mg⋅l^–1^⋅h^–1^ (Kenny et al., 2012).

EG could be metabolized by many kinds of microorganisms through two different pathways (Figure 2). In the pathway of acetogens, EG is degraded to ethanol and acetaldehyde, which is eventually transformed to acetate via acetyl-CoA (Trifunović et al., 2016). In contrast, through the pathway of *Pseudomonas aeruginosa*, EG is initially oxidized into glycolate by a series of dehydrogenases, and the generated glycolate will be further transformed into glyoxylate by the glycolate oxidase (GlcDEF). Glyoxylate could be converted into glycerate, which finally forms pyruvate (Figure 2; Child and Willetts, 1978; Kataoka et al., 2001).

*P. putida* KT2440 could accumulate mclPHA under nitrogen-limiting conditions but could not efficiently utilize EG as its sole carbon source. Through adaptive laboratory evolution, one mutant of *P. putida* KT2440 that could utilize EG as its sole carbon source was isolated. Comparative genomic analyses between the wild strain and the mutant revealed that a transcriptional regulator, GclR, played a central role in repressing the glyoxylate carboligase pathway (Li et al., 2019). With this knowledge, Franden et al. (2018) demonstrated that the overexpression of a combination of the glyoxylate carboligase (Gcl) operon with the glycolate oxidase (GlcDEF) operon endowed *P. putida* KT2440 with the ability to utilize EG as a sole carbon source for growth and accumulate in nitrogen-limiting M9 medium.

As for 6-hydroxyhexanoate, the hydrolysis product of PCL-based PUR, it is first converted to 6-oxohexanoic by the 6-hydroxyhexanoate dehydrogenase (ChnD). 6-oxohexanoic is eventually transformed into adipate by the action of the 6-oxohexanoic dehydrogenase (ChnE). After ligation with CoA, the adipate will be further converted to 3-oxoadipyl-CoA, which finally forms succinyl-CoA and acetyl-CoA that enter the TCA cycle with the production of succinic acid (Figure 2; Brzostowicz et al., 2003).

### From Aromatic Hydrocarbons to Succinic Acids and PHA

Styrene, the aromatic monomer of PS, could be generated from the PS pyrolysis in the absence of air (Kaminsky and Kim, 1999) and is directly utilized as a carbon source by many microorganisms via two different catabolic pathways (Figure 2; O’Leary et al., 2002).

The first one is the direct aromatic ring cleavage pathway (Figure 2). In this pathway, the aromatic ring of styrene is firstly hydroxylated to styrene *cis*-glycol by styrene dioxygenase (SDO). Styrene *cis*-glycol is then further oxidized by a *cis*-glycol dehydrogenase (CGDH) to form 3-vinylcatechol. This product is degraded into pyruvate, which is further converted to acetyl-CoA by the pyruvate dehydrogenase complex (PDHC). Acetyl-CoA will finally enter the TCA cycle to generate succinic acid or be transformed into acetoacetyl-CoA, which could form β-D-Hydroxybutyryl-CoA or could be converted to PHA by an acetoacetyl-CoA reductase (PhaB) or a PHA synthase (PhaC), respectively (Anderson and Dawes, 1990). The other styrene metabolism pathway involves vinyl side-chain oxidation (Figure 2). Styrene is first converted into phenylacetic acid (PAA) by several enzymes, such as styrene monooxygenase (SMO), styrene oxide isomerase (SOI), and phenylacetaldehyde dehydrogenase (PAALDH). PAA is further hydroxylated and passed through the β-oxidation process to yield acetyl-CoA, which will then enter the TCA cycle or be converted into PHA (O’Leary et al., 2005; Oelschlägel et al., 2018).

In 2005, Ward et al. first found that *Pseudomonas putida* CA-3 could convert the metabolite of styrene, PAA, into polyhydroxyalkanoate (PHA) when a limiting concentration of nitrogen was added to the growth medium. Their finding built the metabolic link between styrene degradation and PHA accumulation in *P. putida* CA-3 and found a trail for the microbial valorization of PS waste into valuable chemicals (Ward et al., 2005; Nikodinovic-Runic et al., 2011).

Afterward, Ward et al. (2006) used the styrene oil, the pyrolysis products of PS waste at 520°C in a fluidized bed reactor, as the sole source of carbon and energy to support the growth and PHA accumulation of *P. putida* CA-3 in the shake flask experiments. In a run, the transformation rate from PS waste to PHA was 10%. In order to improve the conversion rate, Goff et al. performed a batch fermentation of *P. putida* CA-3 grown on styrene oil in a stirred tank reactor with an optimized nitrogen feeding strategy (Goff et al., 2007).

### From Aliphatic Hydrocarbons to Fatty Acids, PHA, and Biosurfactants

Although PE, PP, and PVC, with similar carbon–carbon backbone chains, have been shown to be degraded by a number of microorganisms, the key depolymerases involved in the degradation process and the resulting depolymerization products remain unknown. However, pyrolysis in the absence of air could be an alternative method that can effectively depolymerize those plastic wastes into low molecular weight aliphatic hydrocarbons (Aguado et al., 2002).

It has been reported that the pyrolytic hydrocarbons of PE can be degraded via a terminal oxidation process similar to the microbial degradation pathway of *n-*alkane (Figure 2; Yoon et al., 2012; Jeon and Kim, 2015, 2016a). This process starts by the oxidation of a terminal methyl group by an alkane hydroxylase (AH) to generate a primary alcohol, which is further oxidized by an alcohol dehydrogenase (AD) to the corresponding aldehyde and finally converted into fatty acids by an aldehyde dehydrogenase (ADL) (Rojo, 2009). Fatty acids are then conjugated to CoA by an acyl-CoA synthase and further processed by β-oxidation to produce acetyl-CoA, L-β-hydroxyacyl-CoA, and *trans*-2-decenoyl-CoA (Figure 2). Acetyl-CoA can enter the TCA cycle to generate succinic acid or acetoacetyl-CoA, which can be finally converted to PHA under the appropriate condition (Anderson and Dawes, 1990). The L-β-hydroxyacyl-CoA is isomerized into D-β-hydroxyacyl-CoA, which can finally be converted into PHA through a PHA synthase (PhaC) (Sabirova et al., 2006). In addition, the *trans*-2-decenoyl-CoA is hydrated by the R-specific enoyl-CoA hydratase (RhlYZ) to form R-3-hydroxydecanoyl-CoA, which then acts as the direct lipid precursor used by the R-3-((R-3-hydroxyalkanoyl)oxy) alkanoic acids (HAA) synthase (RhlA) for the synthesis of HAAs. HAA, combined with dTDP-L-rhamnoses, can be converted into rhamnolipid biosurfactants by the rhamnosyltransferase 1 (RhlB) and the rhamnosyltransferase 2 (RhlC) (Abdel-Mawgoud et al., 2014).

Guzik et al. (2014) first used the pyrolytic hydrocarbons of PE as the starting material for microbial fermentation to produce PHA. *Pseudomonas aeruginosa* PAO-1, tested from 23 bacterial strains capable of degrading hydrocarbons or producing PHA, was reported to be able to accumulate PHA with almost 25% of cell dry weight when supplied with PE pyrolytic hydrocarbons and biosurfactants. Another bacterial strain, *Ralstonia eutropha* H16 (previously known as *Cuprivadus necator* or *Wausternia eutropha*), also exhibited PHA accumulation when supplied with non-oxygenated PE pyrolytic hydrocarbons as a carbon source in a nitrogen-rich tryptone soya broth (TSB) growth medium (Johnston et al., 2017). In contrast to PE pyrolysis in the absence of air, pyrolysis in the presence of air would not only cleave the long chains of PE but also introduce the carbonyl and hydroxyl groups into the backbone of pyrolytic hydrocarbons, which could improve the bioavailability of pyrolytic hydrocarbons as a carbon source for microbial fermentation to produce PHA by the strain *Ralstonia eutropha* H16 (Radecka et al., 2016).

In addition, PP could also be depolymerized into branched chain fatty alcohols and alkenes by pyrolysis. In 2019, Mihreteab et al. reported that strain *Yarrowia lipolytica* 78-003 was able to convert such depolymerization products to value-added fatty acids when mixed with biosurfactants and trace nutrients. During a period of 312 h, *Y. lipolytica* 78-003 assimilated more than 80% of the substrate and produced up to 492 mg L^–1^ lipids mainly composed of C_16_-C_18_ unsaturated fatty acids (Mihreteab et al., 2019). Johnston et al. (2019) found that *R. eutropha* H16 could utilize oxidized PP fragments as an additional carbon source to produce PHA in TSB medium.

As for PVC, although the pyrolysis at 300°C in the N_2_ flow has been showed to be able to depolymerize PVC into hydrocarbons along with the dechlorination in the form of HCl (Yuan et al., 2014), there are no reports about microbial strains that can utilize PVC pyrolysis products as carbon source so far. However, as these products are of similar chemical compositions to those of PE and PP, it is justifiable to believe that the strains of *P. aeruginosa*, *R. eutropha* H16, and *Yarrowia lipolytica* 78-003, which are able to assimilate the pyrolysis hydrocarbons of PE and PP, could also utilize PVC pyrolysis products to produce valuable chemicals.

Microbial growth on hydrocarbons is often associated with the production of biosurfactant, which can emulsify the hydrophobic hydrocarbons in aqueous media to increase the bioavailability of hydrocarbons to the cells. For instance, the strain *Renibacterium salmoninarum* 27BN was found to be able to produce rhamnolipids when grown on *n*-hexadecane (Christova et al., 2004). An oil-degrading bacterium *Dietzia maris* As-13-3, isolated from deep sea hydrothermal field, could also produce di-rhamnolipid as a biosurfactant, while tetradecane, *n*-hexadecane, and pristine were utilized as sole carbon sources (Wang et al., 2014). These results imply that pyrolysis hydrocarbons of PE, PP, and PVC could also be utilized as feedstocks to produce biosurfactants by these known hydrocarbon-degrading and biosurfactant-producing microorganisms.

## Concluding Remarks and Future Prospects

As described above, a number of plastic-degrading microorganisms and enzymes have been sourced from the environment. However, an understanding of depolymerases contributing to the breakdown of plastics remains scarce. Therefore, future efforts should be devoted to identifying more depolymerases from the plastic-degrading microorganisms. In addition, enhancing the efficiency of enzymatic degradation is a big challenge. On the one hand, the macromolecular aggregate structures of plastics, such as the crystalline structures and cross-linking networks, impede the enzymatic degradation. The development of physical pretreatments, such as mechanical grinding and γ-irradiations, may help disorder these macromolecular aggregate structures and improve enzymatic degradation (Alariqi et al., 2006). On the other hand, approaches of rational protein engineering and direction evolution are necessary to improve the activity and stability of depolymerases, which will benefit the enhancement of enzymatic degradation efficiency.

While the long-chain polymer molecules could have been effectively depolymerized into small subunits (monomers or oligomers) by depolymerases, these small depolymerization products would be incorporated into cells as the feedstocks for metabolism (Table 8). Based on the advances in the understanding of the depolymerases and the microbial metabolic pathways of depolymerization products, it is fascinating to apply synthetic biology to build microbial cell factories that could depolymerize plastic wastes and utilize the small depolymerization products to produce chemicals with high value (Wierckx et al., 2015; Salvador et al., 2019; Blank et al., 2020). If this is manageable, it would not only contribute to the disposal of plastic wastes but also establish an improved cyclic utilization of plastics.

**TABLE 8:** Strains for the valorization of depolymerization products of plastics.

Plastics	Depolymerization methods	Depolymerization products	Strains	Metabolites	Yields	References
PET	Pyrolysis at 450°C	TPA	*Pseudomonas putida* GO16, *Pseudomonas putida* GO19, *Pseudomonas frederiksbergensis* GO23	PHA	8.4 mg⋅l^–1^⋅h^–1^	Kenny et al., 2008
PET	Pyrolysis	TPA	*Pseudomonas putida* GO16	PHA	108.8 mg⋅l^–1^⋅h^–1^	Kenny et al., 2012
PET	–	EG	*Pseudomonas putida* KT2440	PHA	0.06 g PHA per g EG	Franden et al., 2018
PS	–	Styrene	*Pseudomonas putida* CA-3	PHA	0.11 g PHA per g carbon	Ward et al., 2005
PS	–	Styrene	*Pseudomonas putida* CA-3	PHA	3.36 g⋅l^–1^	Nikodinovic-Runic et al., 2011
PS	Pyrolysis at 520°C	Styrene	*Pseudomonas putida* CA-3	PHA	62.5 mg PHA per g styrene	Ward et al., 2006
PS	Pyrolysis	Styrene	*Pseudomonas putida* CA-3	PHA	0.28 g PHA per g styrene	Goff et al., 2007
PE	Pyrolysis	Paraffins from C8 to C32	*Pseudomonas aeruginosa* PAO-1	PHA	25% of the cell dry weight	Guzik et al., 2014
PE	Pyrolysis	Hydrocarbons	*Ralstonia eutropha* H16	PHA	0.46 g⋅l^–1^	Johnston et al., 2017
PE	Pyrolysis in air	Oxidized hydrocarbons	*Ralstonia eutropha* H16	PHA	1.24 g⋅l^–1^	Radecka et al., 2016
PP	Pyrolysis at 540°C	Branched chain fatty alcohols and alkenes	*Yarrowia lipolytica* 78-003	Fatty acids	492 mg⋅l^–1^ over 312 h	Mihreteab et al., 2019
PP	Thermal oxidation at 80–100°C in the oxygen–ozone mixture	Oxidized PP fragments	*Ralstonia eutropha* H16	PHA	1.36 g⋅l^–1^	Johnston et al., 2019
–	–	n-hexadecane	*Renibacterium salmoninarum* 27BN	Rhamnolipid	0.92 g⋅l^–1^	Christova et al., 2004
–	–	n-hexadecane	*Dietzia maris* As-13-3	Di-rhamnolipid	120 mg⋅l^–1^	Wang et al., 2014

## Author Contributions

YY generated the idea and designed the project. YH contributed to the analysis and discussion. JR and YY prepared the figures and tables. YY and JR wrote the manuscript.

## Conflict of Interest

The authors declare that the research was conducted in the absence of any commercial or financial relationships that could be construed as a potential conflict of interest.
